# Optimizing anti-thymocyte globulin dosing in allogeneic hematopoietic stem cell transplantation: individualized approaches and clinical implications

**DOI:** 10.3389/fimmu.2025.1634157

**Published:** 2025-08-08

**Authors:** Haitao Wang, Hongqi Yang, Jishan Du, Liping Dou, Daihong Liu

**Affiliations:** ^1^ Senior Department of Hematology, The Fifth Medical Center of Chinese PLA General Hospital, Beijing, China; ^2^ Translational Medicine Research Center, Medical Innovation Research Division of The Fourth Medical Center of Chinese PLA General Hospital, Beijing, China; ^3^ Department of Emergency Medicine, The Second Medical Center of Chinese PLA General Hospital, Beijing, China; ^4^ Department of Internal Medicine, Medical School of Chinese PLA, Beijing, China

**Keywords:** antithymocyte globulin, graft-versus-host disease, hematopoietic stem cell transplantation, precision dosing, therapeutic drug monitoring, pharmacokinetics

## Abstract

Allogeneic hematopoietic stem cell transplantation (allo-HSCT) is a potentially curative therapy for hematologic malignancies. However, the initial clinical experience with allo-HSCT revealed a concerning prevalence of severe graft-versus-host disease (GVHD) and graft failure. Subsequent randomized studies highlighted the role of anti-thymocyte globulin (ATG) in reducing acute and chronic GVHD and graft failure, although it did not improve overall survival. Pharmacodynamic studies have established an association between ATG concentration and the incidence of GVHD and life-threatening infections. However, ATG concentration at designated timepoints showed no such correlations with non-relapse mortality and overall survival in allo-HSCT. There is a delicate balance between ATG exposure and the outcomes of allo-HSCT. More specifically, insufficient ATG exposure may diminish its function on GVHD prophylaxis, while excessive ATG may delay immune reconstitution and increase risk of disease relapse and infection. Considering the significant inter-individual heterogeneity in ATG pharmacokinetics, individualized ATG dosing could potentially increase the proportion of transplant recipients attaining the optimal ATG exposure. Recent studies have shown that individualized ATG dosing, guided by absolute lymphocyte count or therapeutic drug monitoring, can improve optimal exposure attainment rate. Which indicated a potential approach to achieve superior transplant outcomes. This review summarizes the advances and the challenges of individualized ATG dosing in allo-HSCT.

## 1 Introduction

Despite advances in chemotherapy and novel cellular therapies, allogeneic hematopoietic stem cell transplantation (allo-HSCT) remains a well-established curative therapy for defined subsets of hematologic malignancies (1, 2). In its initial historical application decades ago, allo-HSCT was associated with substantial risks, including graft-versus-host disease (GVHD) and graft failure (GF) (3, 4). Multiple randomized trials demonstrated that anti-thymocyte globulin (ATG) could reduce the incidence of both severe acute (aGVHD) and chronic GVHD (cGVHD) post-transplant (5–7), while some randomized studies reported no significant improvement in cGVHD-free survival in specific cohorts with ATG (8, 9). Collectively, these findings provide a compelling rationale for the incorporation of ATG into allo-HSCT to prevent GVHD.

ATG is a polyclonal antibody that could deplete a variety of immune cells, while the primary mechanism of GVHD prophylaxis is T-cell depletion (10). Historically, three main types of ATG products have been available for clinical use. The first ATG formulation was horse-derived ATG (ATGAM^®^, Pfizer, USA) (11). ATGAM^®^ is not typically used for the indication of allo-HSCT, as two prospective trials failed to demonstrate its efficacy in prophylaxis of aGVHD (12, 13). The other two ATGs, Thymoglobulin^®^ (ATG-T, Sanofi, France) and Grafalon^®^ (formerly known as ATG-Fresenius, ATG-F, Neovii, Germany), are both derived from rabbits. Although most of these products are commercially available, ATG-T remains the most commonly used ATG preparation in clinical practice (14, 15). Consequently, this review will focus on the investigations into optimizing the dosage of ATG-T. It is important to note that there is no universally accepted bioequivalent dosing between ATG-T and ATG-F, special caution should be exercised when switching between the two ATG preparations in clinical practice (16–18).

Pharmacological studies of ATG found that the immunological effects of ATG are critically influenced by its concentration (19–26). Therefore, optimizing ATG dose in allo-HSCT to maximize its GVHD prophylaxis effect and minimize its potential side effects is crucial for improving transplant outcomes (14, 27, 28). Early studies explored the optimal ATG dose using body weight-adjusted dosing strategy (6, 8, 29). However, due to ATG pharmacokinetics being influenced by body weight of recipients, lymphocyte count and timing of ATG administration, the inter-individual heterogeneity is considerable (30–32). As such, the optimal ATG dose in allo-HSCT has not yet been determined. Given the ATG pharmacokinetic heterogeneity among transplant recipients, individualized ATG dosing may be a potential solution and has garnered significant research interest. Recent pharmacological studies have found that optimal ATG exposure is associated with lower incidence of GVHD and virus reactivation, and may even lead to improved non-relapse mortality (NRM) and overall survival (OS) (33–35). Importantly, achieving optimal ATG exposure through individualized dosing can reduce adverse events in allo-HSCT and improve health-related quality of life (19, 36, 37).

This review aims to provide a comprehensive summary of the advances of individualized ATG dosing in allo-HSCT and its effect on transplant outcomes.

## 2 Immunomodulatory effects and concentration detection of ATG

**Figure 1:**
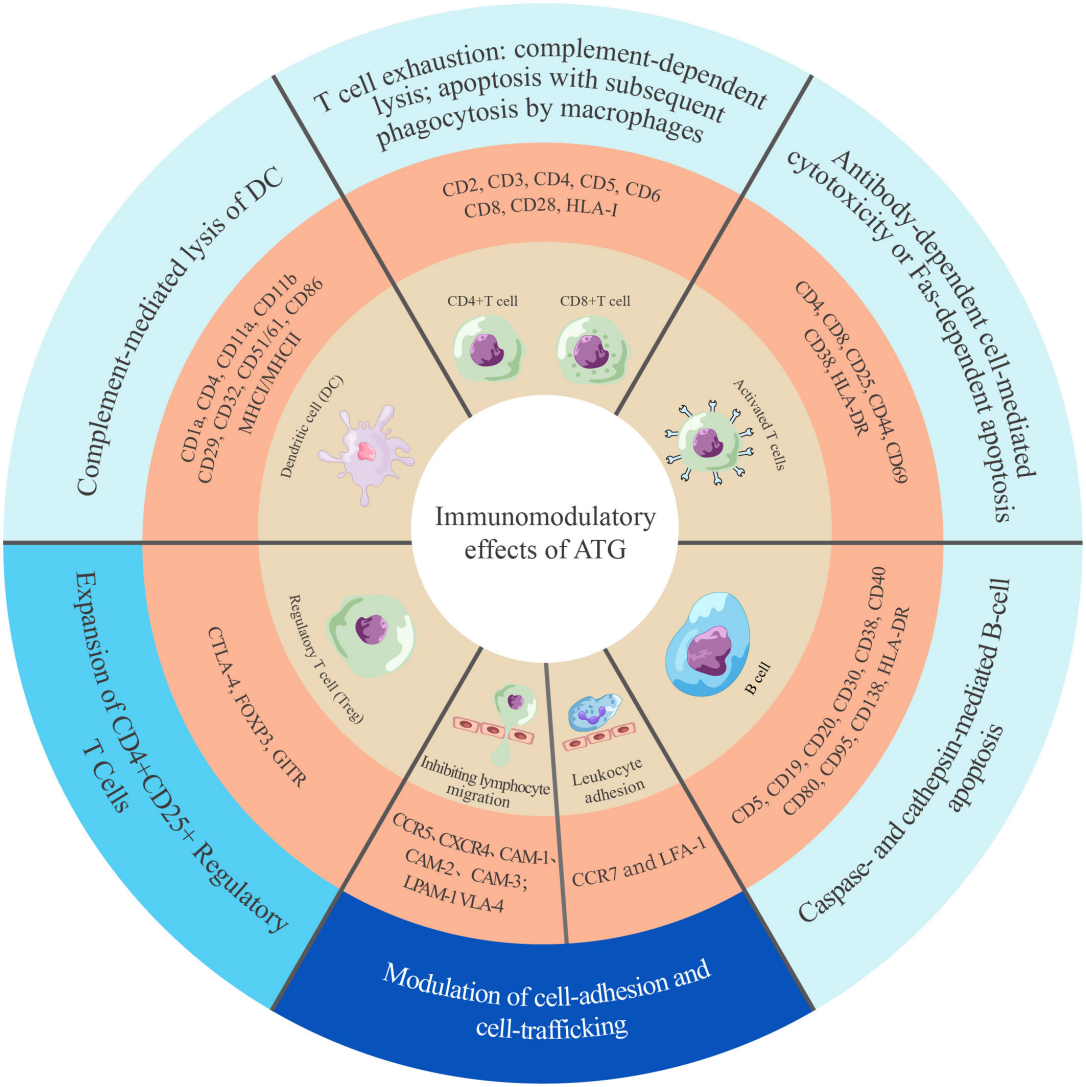
Landscape of ATG-induced immunomodulation mechanisms. The mechanisms are categorized into three groups, indicated by colors in the outermost circle: cell clearance and apoptosis (light blue) (14, 38, 41, 42), cell expansion (lake blue) (43), and cell adhesion and trafficking (dark blue) (45). CAM, cell adhesion molecule; CCR, C-C chemokine receptor; CD, cluster of differentiation; CTLA, cytotoxic T-lymphocyte antigen; CXCR, C-X-C chemokine receptor; DC, dentritic cell; FOXP3, forkhead box P3; GITR, glucocorticoid-induced tumor necrosis factor receptor family-related protein; HLA-DR, human leukocyte antigen-DR isotype; HLA-I/II, human leukocyte antigen class I/II; LFA, lymphocyte function-associated antigen; LPAM, lymphocyte Peyer’s patch adhesion molecule; VLA, very late antigen.

ATG-T is a heterologous polyclonal immunoglobulin G (IgG) that targets over 40 antigens (14, 15). These antigens are classified into two categories based on their biological function: immune cell response antigens and adhesion/cell-trafficking molecules (**Figure 1**) (14, 15, 38, 39). ATG-T mediates its immunomodulatory effects primarily by targeting T cells and other immune effector cells. It targets key T-cell antigens, including CD2, CD3, CD4, CD5, CD6, CD8, CD28 and HLA class I molecules, leading to T-cell depletion via complement-dependent lysis and T-cell activation-induced apoptosis (38, 40). Additionally, ATG-T also contains antibodies against B-cell surface proteins CD5, CD19, CD20, CD30, CD38, CD40, CD80, CD95, CD138 and HLA-DR, triggering caspase- and cathepsin-dependent B cell apoptosis (38, 40, 41). Furthermore, ATG-T could inhibit dendritic cell (DC) maturation and migration by targeting CD1a, CD4, CD11a, CD11b, CD29, CD32, CD51/61, CD86, MHC I and MHC II (38, 42). *In vitro* studies have demonstrated its capacity to expand CD4+ CD25+ regulatory T cells (Tregs) by targeting CTLA-4, FOXP3, GITR (43, 44). Finally, ATG modulates leukocyte-endothelial interactions by targeting integrins (VLA-4, LPAM-1), chemokine receptors (CXCR4, CCR5, CCR7), and leukocyte adhesion molecules (ICAM-1, ICAM-2, ICAM-3), thereby disrupting leukocyte adhesion to endothelia (38, 45).

It is important to note that the immunomodulatory effects of ATG depended critically on its concentration. Specifically, a low dose of ATG (e.g., 1 mg/kg) is sufficient to induce antibody-dependent cell-mediated cytotoxicity (ADCC) against activated T cells in blood circulation. However, this ATG concentration is inadequate for depleting lymphocytes (T cells, B cells and NK cells) and antigen-presenting cells residing within secondary lymphoid tissues (22, 46). Additionally, B cells (CD20+) and NK cells (CD16+/CD56+) may only be affected at higher doses (> 5mg/kg) of ATG-T (22). Lower-dose ATG selectively depleted activated T cells while preserving the function of B and NK cells, thereby mitigating systemic immunosuppression (14). Although the effects of ATG are dose-dependent on various cell types, it needs special caution to adjust the dose of ATG for individuals to improve the efficacy of HSCT.

The concentration of ATG, often labeled on the vial, generally refers to the total ATG. Total ATG levels in patient samples could be quantified by enzyme-linked immunosorbent assay (ELISA) (47, 48). The component capable of binding to human lymphocytes was defined as active ATG. Despite comprising only 10% of total ATG, active ATG significantly affects aGVHD, immune reconstitution and post-transplant lymphoproliferative disorder (PTLD) (19–21, 49). The quantification of active ATG remains challenging (50, 51), with flow cytometry being the most widely utilized method for its detection (21, 51). In 2020, liquid chromatography-mass spectrometry (LC-MS) was employed for the first time to quantify the active fraction of ATG in plasma (52). This technique offers superior precision; however, its application remains limited due to restricted accessibility. The establishment of ATG detecting methods enables its pharmacokinetic and pharmacodynamic evaluation in allo-HSCT (**Table 1**).

**Table 1:** Systematic comparison of three ATG quantification methods.

Method	Detected ATG component	Detection platform	Sample volume required for single detection	Fluorescent labeling of antibody	Lymphocytes as vectors	High-throughput detection (YES/NO)
ELISA (47, 48)	Total ATG	Enzyme immunoassay	20-100μL	HRP	Not required	YES
Flow Cytometry (21, 51)	Active ATG	Flow cytometer	50-100μL	FITC or PE	Required	NO
LC-MS/MS (52)	Total and active ATG	LC-MS System	10μL	No antibody	Required when detecting active ATG	YES

ATG, anti-thymocyte globulin; ELISA, enzyme-linked immunosorbent assay; FITC, fluorescein isothiocyanate; HRP, horseradish peroxidase; LC-MS, liquid chromatography-mass spectrometry; MS, mass spectrometry.

## 3 Interindividual heterogeneity in ATG pharmacokinetics

The complex immunomodulatory mechanisms of ATG underlies the significant interindividual heterogeneity in its pharmacokinetics (27, 48, 51, 53–55). *Waller*, et al. (48) reported that the clearance of ATG was relatively slow, and serum total ATG remained detectable up to 90 days post-transplant. The calculated half-life of active and total ATG were 7 days and 14 days, respectively. The study further demonstrated that the time for active ATG levels decreasing to sub-therapeutic levels (1 μg/mL) in the 6 mg/kg group (17 days) was significantly shorter than 10 mg/kg group (45 days; *P* = 0.002). Similarly, when using 16-20mg/kg ATG-T, the median time for active ATG level to decline to less than 2.0μg/ml was 45.5 days (51). The clearance time of the 16–20 mg/kg ATG group was not significantly longer than that of the 10 mg/kg group, suggesting that a higher dose (> 10 mg/kg) of ATG-T may not be necessary. An Austrian study by *Seidel*, et al. found that the half-life of ATG-T was consistent when the ATG-T dose within the range of 7.5-20mg/kg, with a linear correlation between the dose and maximum serum concentration (C_max_). However, when the ATG-T dose was 30–40 mg/kg, the active fraction of ATG-T accumulated in the body, leading to a sharp increase in C_max_ and resulting in ATG overexposure (55).

**Figure 2:**
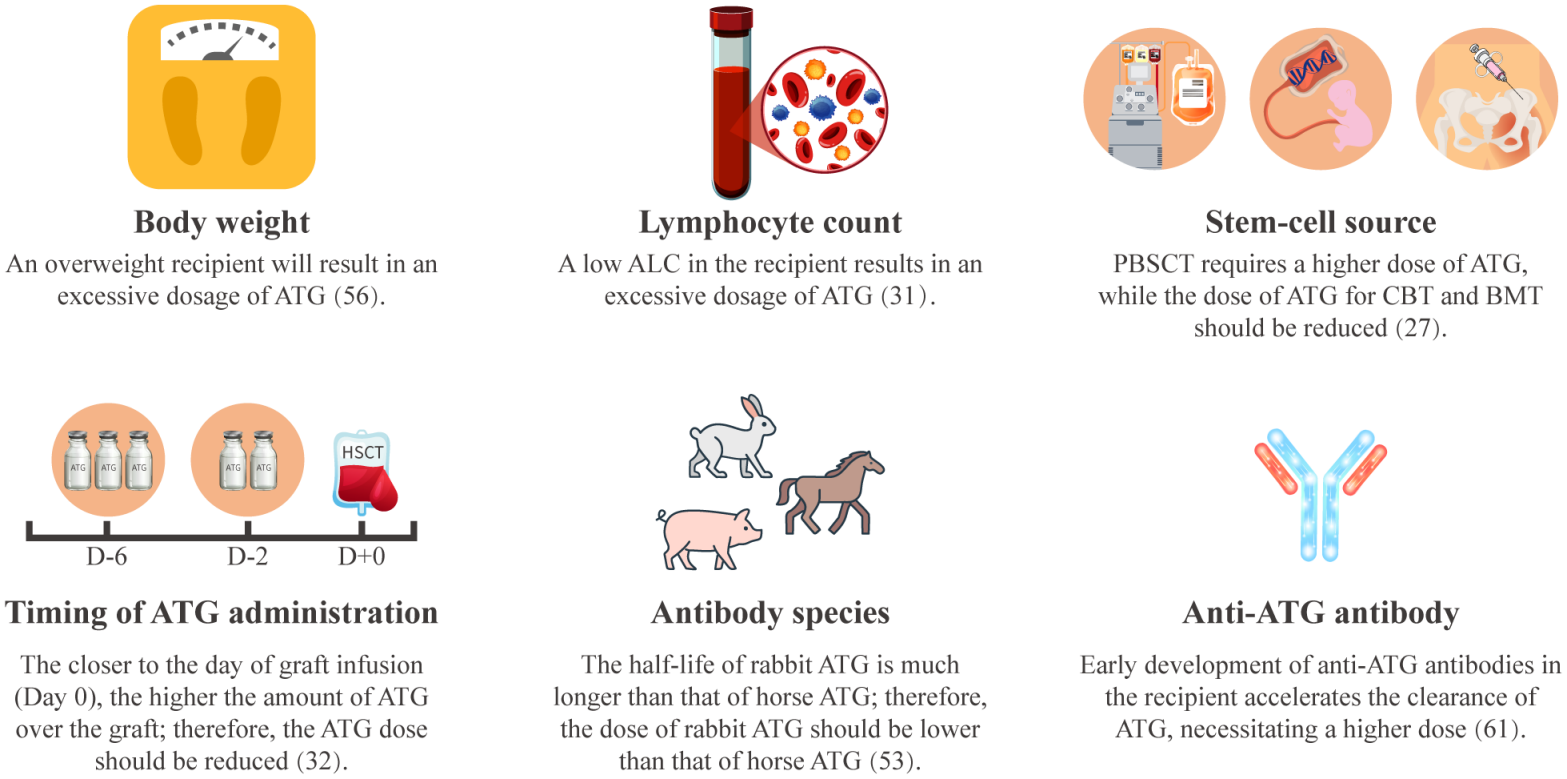
Factors influencing ATG pharmacokinetics. ALC, absolute lymphocyte count; ATG, anti-thymocyte globulin; BMT, bone marrow transplantation; CBT, cord blood transplantation; PBSCT, peripheral blood stem cell transplantation.

Weight-based ATG dosing induces marked interindividual variability in ATG exposure, arising from recipient-specific and regimen-related determinants (**Figure 2**). Body weight and absolute lymphocyte count (ALC) constitute principal recipient-specific determinants of ATG clearance. Pharmacokinetic analyses demonstrate that pediatric HSCT recipients with higher body weight and lower ALC exhibit over exposure to active ATG (31, 56).

Both graft source and timing of ATG administration significantly modulate ATG exposure (27, 32). Compared to G-CSF-mobilized peripheral blood stem cells (G-PBSC), bone marrow and cord blood grafts contain fewer memory T cells and more naïve T cells, contributing to delayed post-transplant T-cell reconstitution. This necessitates ATG dose reduction in bone marrow or cord blood HSCT to promote T-cell recovery (32, 57–59). The timing of ATG administration is also important. Early ATG administration (between days -9 and -5) demonstrated reduced ATG exposure and accelerate T-cell reconstitution compared to later administration (between days -5 and 0) (32, 60).

Furthermore, ATG pharmacokinetics differ between preparations. Rabbit-ATG (Thymoglobulin^®^) exhibits a longer half-life, with detectable plasma active ATG persisting for one month, whereas active horse-ATG (ATGAM^®^) components decline within two weeks (53). As xenogeneic proteins, ATG preparations can induce anti-ATG antibodies. Early antibody formation (before day +22) mediates accelerated ATG clearance, substantially reducing post-transplant exposure (61).

## 4 ATG dose adjustment guided by ATG concentration at designated timepoints or GVHD biomarkers

### 4.1 ATG dose adjustment guided by ATG concentration at designated timepoints

Several studies have highlighted the association between ATG concentrations at designated timepoints of allo-HSCT and transplant outcomes (**Table 2**). Generally, increased ATG concentrations reduce the risk of GVHD, while most studies suggest that ATG concentrations at designated timepoints do not affect the incidence of relapse, death, or infection. A study conducted by *Remberger*, et al. in Sweden reported that patients with serum ATG-T levels >70 μg/mL on day 0 had lower risk of grades II-IV aGVHD compared to those with ATG-T levels <70 μg/mL (11% vs 48%, *P* = 0.006) (47). In another study by the same group, patients received ATG-T at a total dose of 6 or 8 mg/kg as part of GVHD prophylaxis. The results revealed that patients with total ATG-T levels ≤ 40 μg/mL on day +11 had a higher incidence of grades III-IV aGVHD (32% vs. 0%, *P* < 0.01). However, their OS and relapse-free survival (RFS) at 5 years were similar (62). *Elmahdi*, et al. from Japan reported that a lower total ATG-T concentration in week 4 post-transplant was an independent risk factor for grade II-IV aGVHD, but no correlation was found between total ATG-T concentration at week 2 or 4 and recurrence (63). *Chawla*, et al. evaluated the relationship between active ATG concentration and GVHD in 180 allo-HSCT recipients receiving 4.5 mg/kg ATG-T. Higher concentrations at days +7 and +28 correlated with a reduced risk of aGVHD, while elevated levels at days 0, + 7, and +28 were associated with lower cGVHD incidence (64). Similarly, *Podgorny*, et al. from Canada found that both higher ATG-T levels on day +7 and +28 were associated with lower risks of grade II-IV aGVHD and cGVHD, but not with relapse, death, or infection (20). *Teramoto* et al. identified ATG concentration on day 0 (C_day_0_) as the strongest predictor for grade II-IV aGVHD. They found C_day_0_ ≥ 20μg/mL correlated with an approximately 3-fold reduced risk of aGVHD and 2-fold decrease in overall mortality and relapse. Their population pharmacokinetic modeling indicated a total ATG dose of 3 mg/kg (1.5 mg/kg per dose on days -2 and -1) to achieve target C_day_0_ with 80% probability (65).

**Table 2:** Association between ATG concentration at designated timepoints and transplant outcomes.

Author	Donor type	Stem cell source	Malignant/Benign	Gender	Children/Adults Age, Median (range)	Conditioning regimen	Total r-ATG dosage	Total/Active r-ATG	Timing of ATG monitoring	Association with clinical outcomes
Remberger, et al. (47)	MUD	BM (n=28) PBSC (n=33)	Malignant (n=53) Benign (n=8)	Male (n=35) Female (n= 26)	Children (n=14). Adults (n=47) 35 (1–61)	TBI-based MAC (n=27) BU-based MAC (n=25) RIC (n=9)	4mg/kg (n=14) 6mg/kg (n=21) 8mg/kg (n=15) 10mg/kg (n=11)	Total r-ATG	Day 0, Week 1, 2, 3, 4, 5	r-ATG > 70 μg/mL vs < 70μg/mL on Day 0: lower risk of developing grade II-IV aGVHD (11% vs 48%, *P* = 0.006). r-ATG >45 μg/mL vs r-ATG < 45μg/mL on Week 1: lower risk of developing grades II-IV aGVHD (18% vs 52%, *P* = 0.01).
Remberger, et al. (62)	MUD (n=5) MMUD (n=38)	CB	Malignant (n=27) Benign (n=16)	Male (n=31) Female (n=12)	Children (n=26). Adults (n=17) 16 (0.4-65)	TBI-based MAC (n=11) BU-based MAC (n=16) RIC (n=16)	6mg/kg (n=27) 8mg/kg (n=16)	Total r-ATG	Day 0, + 11, + 25	r-ATG ≤ 40 μg/mL vs > 40μg/mL on Day +11: higher incidence of grade III–IV aGVHD (32% vs. 0%, p<0.01), higher TRM (69% vs. 7%, *P* = 0.005), less relapse (17% vs. 82%, *P* < 0.01).
Elmahdi, et al. (63)	MUD (n=8), MMUD (n=10) MMRD (n=19)	BM (n=20) CB (n=2) BM + PBSC (n=15)	Malignant (n=14) Benign (n=23)	Male (n=17) Female (n=20)	Children (n=35). Adults (n=2) 8 (1-19)	TBI-based MAC (n=36) TLI-based MAC (n=1)	10mg/kg (n=21) 15mg/kg (n=16)	Total r-ATG	Week 2 and 4	Grade II-IV aGVHD: lower r-ATG levels (*P* = 0.004). r-ATG < 6.2μg/mL at Week 4: an independent risk factor for grade II-IV aGVHD (*P* = 0.037).
Chawla, et al. (64)	MSD (n=67) Other (n=113)	PBSC	Malignant	Male (n=104) Female (n=76)	Adults 50 (18-66)	BU-based MAC (n=177) Other (n=3)	4.5mg/kg	Active r-ATG	Day 0, + 7, + 28	High active ATG on day +7 and +28: lower risk of aGVHD. High active ATG on day 0, + 7, and +28: reduced risk of cGVHD.
Podgorny, et al. (20)	MSD (n=76) MUD (n=51) HLA-mismatched (n=26)	BM (n=10) PBSC (n=143)	Malignant (n=147) Benign (n=6)	Male (n=91) Female (n=62)	Adults 49 (19-66)	MAC with TBI (n=96) MAC without TBI (n=57)	4.5mg/kg	Active r-ATG	Day +7 and +28	Active ATG > 1.454 mg/L vs < 1.454 mg/L on Day +7: 0.35-fold risk of grade II-IV aGVHD (*P* = 0.019) Active ATG > 0.029 mg/L vs < 0.029 mg/L on Day +28: 0.52-fold risk of grade II-IV aGVHD (*P* = 0.002) Active ATG > 0.803 mg/L vs < 0.803 mg/L on Day +7: 0.52-fold risk of cGVHD (*P* = 0.025) Active ATG > 0.052 mg/L vs < 0.052 mg/L on Day +28: 0.58-fold risk of cGVHD (*P* = 0.019) Active ATG > 1.436 mg/L vs < 1.436 mg/L on Day +7: 5.84-fold risk of PTLD (*P* = 0.039) Active ATG > 0.082 mg/L vs < 0.082 mg/L on Day +28: 6.63-fold risk of PTLD (*P* = 0.014)
Teramoto et al. (65)	Related (n=99), Unrelated (n=4)	PBSC	Malignant	Male (n=67) Female (n=36)	Adults 47 (17-70)	TBI-based MAC	2.5 mg/kg (n=92) 3 mg/kg (n=11)	Total r-ATG	Day 0	Day 0 r-ATG concentrations ≥ 20 µg/mL are associated with a ∼3-fold reduced risk of Grade II–V aGVHD (HR = 0.32, 95% CI 0.16–0.62) and a ∼2-fold lower risk of overall mortality (HR = 0.47, 95% CI 0.28–0.77) and relapse (HR = 0.50, 95% CI 0.26–0.94).
Jol-van der Zijde, et al. (61)	MSD (n=8) MUD (n=46) MMUD (n=18)	BM (n=44) PBSC (n=16) CB (n=12)	Malignant (n=43) Benign (n=29)	NA	Children Anti-ATG (n=20): 9.6 (1.7-17.0) No anti-ATG (n=52): 5.0 (0.6-17.7)	TBI-based MAC (n=30) Non-TBI based (n=42)	10mg/kg	Total and active r-ATG	pre-HSCT, at least once a week until week 4, and once every 2 weeks until week 13 post HSCT	Early (day 16-22) vs Late (day 28-46) IgG anti-ATG: higher incidence of grade II-IV aGVHD (75% vs 17%) Anti-ATG vs No anti-ATG: higher incidence of grade II-IV aGVHD (35% vs 10%, *P* = 0.01)

aGVHD, acute GVHD; ATG, anti-thymocyte globulin; BM, bone marrow; BU, busulfan; CB, cord blood; cGVHD, chronic GVHD; CI, confidence interval; GVHD, graft-versus-host disease; HLA, human leukocyte antigen; HR, hazard ratio; HSCT, hematopoietic stem cell transplantation; MAC, myeloablative conditioning; MMRD, mismatched related donor; MMUD, mismatched unrelated donor; MRD, matched related donor; MSD, matched sibling donor; MUD, matched unrelated donor; NA, not applicable; PBSC, peripheral blood stem cell; PTLD, posttransplant lymphoproliferative disorder; r-ATG, rabbit ATG; RIC, reduced intensity conditioning; TBI, total body irradiation.

To investigate the reason why ATG concentration at designated timepoints did not affect transplant outcomes, *Jol-van der Zijde*, et al. measured concentrations of ATG-T and anti-ATG antibodies in pediatric HSCT recipients. They found that 28% of the recipients developed anti-ATG antibodies. Early production of these antibodies (before day +22 of HSCT) led to a rapid decrease in ATG concentration and swift recovery of T cells (61). These findings suggest that overall ATG exposure is more important than concentration at a designated timepoint.

### 4.2 ATG dose adjustment guided by GVHD biomarkers

Several studies have investigated biomarker-guided individualized ATG dosing to optimize allo-HSCT outcomes (**Table 3**) (66–69). As early as 2001, Bacigalupo et al. demonstrated the efficacy of this approach in alternative donor bone marrow transplantation. Patients with serum bilirubin levels ≥ 0.9 mg/dl and blood urea nitrogen (BUN) ≥ 21 mg/dl on day +7 were defined as a high-risk group. An additional dose of 3.75 mg/kg ATG-T (1.25 mg/kg on days +7, +9, and +11) was added to high-risk patients. This intervention significantly reduced severe GVHD from 55% to 27% and 1-year transplant-related mortality (TRM) from 60% to 40% (66). A subsequent multicenter randomized trial confirmed these findings: the same ATG regimen significantly reduced grade III–IV aGVHD (15% to 5%) and cGVHD (26% to 11%) in high-risk recipients, though it demonstrated no significant benefit for TRM or OS (67).

**Table 3:** Individualized ATG dosing guided by GVHD biomarkers in clinical trials.

Author	Donor type	Stem cell source	Malignant/Benign	Children/Adults	Conditioning regimen	Total r-ATG dosage	Timing of ATG measurement	Association with clinical outcomes
Bacigalupo, et al. (66)	MUD (n=109)	BM	Malignant	Adults (95%)	TBI-based MAC	7.5mg/kg (n=29) 15mg/kg (n=27)	7.5mg/kg: Day -4 and -3; 15mg/kg: Day -5, -4, -3, and -2	Reduction in GVHD grade III-IV (only in 15 mg/kg group) from 50% to 11%, (*P* = 0.001), reduction in cGVHD from 62% to 39%, (p=0.04), no reduction in TRM.
Bacigalupo, et al. (67)	Related (n=25) Unrelated (n=145)	BM (n=134) PBSC (n=36)	Malignant	Adults	TBI-based MAC (n=98) RIC (n=72)	7.5mg/kg (n=86) 10mg/kg (n=84)	7.5mg/kg: Day-3, -2 10mg/kg: Day-3, -2 +7, +9	Reduced aGVHD (Grade III-IV) from 15% to 5% (*P* = 0.02), reduced cGVHD from 26% to 11% (*P* = 0.03), no significant TRM reduction
Khanolkar, et al. (68)	MSD (n=74) MUD (n=97) MMUD(n=40)	PBSC	Malignant	Adults	TBI-based MAC	4.5mg/kg (n=143) 7.5mg/kg (n=68)	4.5mg/kg: Day-2, -1, 0 7.5mg/kg: Day-2, -1, 0, + 8	Reduction in sGVHD in high-risk trial patients (HR = 0.48, p < 0.05), no significant change in overall survival, and increased non-GVHD-associated NRM due to infections (HR = 3.73, *P* < 0.05).
Xue, et al. (69)	MRD (n=11) MUD(n=16) MMUD (n=11) MMRD(n=25)	PBSC	Malignant	Adults	MAC (n=43) RIC (n=20)	5mg/kg (n=21)	Day +5	Delayed platelet engraftment (29% vs. 45% at 30 days, *P* = 0.03), reduced incidence of cGVHD at 1 year (15% vs 41%, p = 0.04), no differences in grade II-IV aGVHD (29% vs 24%, *P* = 0.86)

aGVHD, acute GVHD; ATG, anti-thymocyte globulin; BM, bone marrow; cGVHD, chronic GVHD; GVHD, graft-versus-host disease; MAC, myeloablative conditioning; MMRD, mismatched related donor; MMUD, mismatched unrelated donor; MRD, matched related donor; MSD, matched sibling donor; MUD, matched unrelated donor; NRM, non-relapse mortality; PBSC, peripheral blood stem cell; r-ATG, rabbit ATG; RIC, reduced intensity conditioning; sGVHD, significant GVHD; sHR, sub-hazard ratio; TBI, total body irradiation; TRM, transplant-related mortality.

In a study of adult peripheral blood stem cell transplantation (PBSCT), *Khanolkar* et al. defined patients with day +7 serum sIL-2Rα levels >4500 ng/L or IL-15 levels <31 ng/L as being at high risk for GVHD. These high-risk patients received an additional dose of 3 mg/kg ATG on day +8, following a conditioning regimen with 4.5 mg/kg ATG. Compared with controls, this strategy significantly reduced the risk of clinically significant GVHD (hazard ratio, 0.48, *P* = 0.045), without increasing relapse. However, the OS benefit was offset by a higher rate of infections in the intervention group, resulting in no improvement in OS (68). More recently, in a study of post-transplant cyclophosphamide (PT-Cy)-based allogeneic PBSCT, *Xue* et al. administered an additional 5 mg/kg anti-T-lymphocyte globulin (ATLG) on day +5 to patients receiving grafts with CD3+ counts > 3 × 10^8^/kg. Compared with historical controls, the addition of ATLG significantly reduced 1-year cGVHD (41% vs. 15%, *P* = 0.04) but did not impact grade II-IV aGVHD, NRM, or OS (69). Consistent with these data, the biomarker-guided personalized ATG dosing strategy ultimately failed to improve patient survival across studies.

## 5 Timing of ATG administration and its impact on transplant outcomes

The timing of ATG administration significantly influences allo-HSCT outcomes. Late ATG administration (closer to day 0) more effectively depletes donor T cells in the graft, while its effect on recipient T cells and antigen-presenting cells remain comparable with earlier dosing. Consequently, late ATG administration is often associated with reduced GVHD but carries an increased risk of viral reactivation compared to early dosing (before day -5) (70).

These timing-dependent effects are further supported by clinical studies. In severe aplastic anemia patients undergoing haplo-PBSCT, *Wu*, et al. demonstrated that shifting ATG dosing from early (days -9 to -7) to late (days -5 to -3) effectively controlled GVHD but led to increased rates of CMV reactivation and EBV-associated post-transplant lymphoproliferative disorder (EBV-PTLD) (71). Conversely, early ATG administration appears to facilitate T-cell reconstitution. *Lindemans*, et al. observed accelerated reconstitution of CD3+, CD4+, and naïve T cells in cord blood transplant recipients receiving early ATG (days -9 to -5) compared to later ATG (days -5 to 0) (32). Similarly, a Japanese study in adult PBSCT found that early ATG administration (1.25mg/kg on day -4), rather than the standard schedule (1.25 mg/kg, days -2 and -1), reduced post-transplant ATG exposure and accelerated CD4+ T-cell recovery (60). These findings indicate that ATG dosing should be adjusted according to timing of administration. A relatively increased dose may be necessary with early ATG dosing, whereas the dose could be reduced if ATG administered closer to day 0.

## 6 Impact of ATG exposure on transplant outcomes

ATG exposure was quantified using the area under the concentration-time curve (AUC). Total ATG exposure was divided into pre- and post-transplant exposure using day 0 (graft infusion) as the reference point. ATG exposure better predicts outcomes in allo-HSCT than the concentration at designated timepoints. Several studies have assessed the association between ATG exposure and transplant outcomes, including GVHD, immune reconstitution, relapse, and survival (**Table 4**) (19, 21, 31, 72–76).

**Table 4:** Association between ATG exposure and transplant outcomes.

Author	Donor type	Stem cell source	Diagnosis (Malignant/Benign)	Gender	Children/Adults	Conditioning regimen	Total r-ATG dosage	Total/Active r-ATG	Pre-/Post-transplant AUC	Association with clinical outcomes
Admiraal, et al. (31)	MUD (n=111) MMUD (n=35)	PBSC	Malignant	Male (n=84) Female (n=62)	Children (n=7) Adult (n=139) 50 (32-59)	Non-MAC	8mg/kg	Active r-ATG	Pre- and Post-transplant AUC	Optimal ATG exposure: 60–95 AU/mL/day 5-year OS: optimum exposure vs above optimum (69% vs 48%, *P* = 0.030), optimum exposure vs below optimum (69% vs 32%, *P* = 0.00037) EFS: below optimum vs optimum exposure (HR 2.54, *P* = 0.007) RRM: above optimum vs optimum exposure (HR 2·66, *P* = 0·027). NRM: below optimum vs optimum exposure (HR 4.36, *P* = 0.004) III-IV aGVHD: below optimum vs optimum exposure (HR 3.09, *P* = 0.029).
Admiraal, et al. (21)	NA	BM (n=118) CB (n=91) PBSC (n=42)	Malignant (n=116) Benign (n=135)	Male (n=157) Female (n=94)	Children and young adult 6.2 (0.2-22.7)	BU-based MAC (n=191) TBI-based MAC (n=54) RIC (n=6)	10mg/kg	Active r-ATG	Pre- and post-transplant AUC	Every 1% increase in post-transplant AUC: decreased CD4+ reconstitution (OR 0.991, *P* < 0.0001) Pre-transplant AUC ≥ 40 AU × day/mL vs < 40 AU × day/mL: lower incidence of grade II-IV aGVHD (HR 0·979, *P* = 0.0081), grade III-IV aGVHD (HR 0·975, *P* = 0.033), cGVHD (HR 0·983, *P* = 0.029) and GF (HR 0·981, *P* = 0.020) Post-transplant AUC in matched BMT or PBSCT < 50 AU × day/mL vs ≥ 50 AU × day/mL: better OS (HR 4.19, *P* = 0.021).
Admiraal, et al. (72)	MUD (n=55) MMUD (n=82)	CB	Malignant (n=56) Benign (n=81)	Male (n=82) Female (n=55)	Children and young adult 7.4 (0.2-22.7)	BU-based MAC (n=122) TBI-based MAC (n=10) Other (n=6)	ATG (n=112) 10mg/kg 7.5mg/kg (BW >40kg, 2010 onwards) No-ATG (n=25)	Active r-ATG	Pre- and post-transplant AUC	Every 10% increase in post-transplant AUC: decreased CD4+ reconstitution (HR= 0.974, P <.0001). Post-transplant AUC > 16 AU × day/mL vs ≤16 AU × day/mL: lower EFS (47% vs 72%, *P* = 0.007).
Jamani, et al. (73)	MRD (n=79) MUD (n=97) MMUD (n=43)	BM (n=6) PBSC (n=213)	Malignant (n=215) Benign (n=4)	NA	Adult 53 (41-60)	BU-based MAC (n=214) Other (n=5)	4.5mg/kg	Active r-ATG	Pre- and post-transplant AUC	Pre-transplant AUC [178 (46-215) mg.hr/L] and post-transplant AUC [588 (198-759) mg.hr/L): higher aGVHD and worse cGRFS.
Oostenbrink et al. (74)	HLA-matched unrelated (n=101)	BM (n=74) PBSC (n=27)	Malignant	Male (n=63) Female (n=38)	Children 9.2 (0.6-18.6)	TBI-based MAC (n=52) Treosulfan-based MAC BU-based (n=33) MAC (n=16)	45mg/kg	Active ATLG	Post-transplant AUC	Prolonged active ATLG exposure (≤16 days) had a lower incidence of aGVHD (50% vs. 8.2%; *P* < 0.001) and an increased risk of relapse in those transplanted in CR2 or 3 (*P* = 0.01).
Dabas, et al. (75)	MSD (n=55), MUD (n=97)	PBSC	Malignant	Male (n=89) Female (n=63)	Adult 53 (18-71)	BU-based MAC	4.5mg/kg	Active r-ATG	Pre- and post-transplant AUC	High pre-transplant AUC of MNC- (> 282.36 UE*hr/L) and CD33+ cells- (> 60.53 UE*hr/L) binding ATG: lower CIR and higher RFS. High post-transplant AUC of lymphocyte-binding ATG (> 1022.42 UE*hr/L): higher CIR and lower RFS. High pre-transplant AUC lymphocyte-binding ATG (> 374.47 UE*hr/L): faster engraftment (*P* = 0.025)
Wang, et al. (19)	HLA-haploidentical	PBSC	Malignant	Male (n=80) Female (n=26)	Children (n=11) Adult (n=95) 32 (14-62)	BU-based MAC (n=93) TBI-based MAC (n=13)	10mg/kg	Active r-ATG	Pre- and post-transplant AUC, Total AUC	Optimal total AUC: 100-148.5 UE/mL/day Optimal vs non-optimal AUC: lower cumulative incidence of CMV reactivation (60.6% vs 77.1%, *P* = 0.016) and persistent EBV viremia (33.1% vs 52.6%, *P* = 0.048), a trend towards improved 2-year OS (75.7% vs 57.8%, *P* = 0.061).
Yang, et al. (76)	HLA-haploidentical	CB	Malignant	NA	NA (n=119)	MAC (n=119)	10mg/kg	Active r-ATG	Post-transplant AUC	Optimal AUC 55–75 AU/mL/day Optimal vs non-optimal AUC: reduced 2-year incidence of relapse (15.2% vs 38.9%, *P* = 0.006), higher LFS (84.8% vs 49.5%, *P* < 0.001), higher OS (89.5% vs 50.8%, *P* = 0.015) higher GRFS (71.3% vs 39.1%, *P* = 0.003)

ATG, anti-thymocyte globulin; AUC, area under the curve; aGVHD, acute GVHD; BM, bone marrow; BU, busulfan; CB, cord blood; cGVHD, chronic GVHD; CIR, cumulative incidence of relapse; CMV, cytomegalovirus; cGRFS, cGVHD- and relapse-free survival; Epstein-Barr virus, Epstein-Barr virus; GF, graft failure; GRFS, GVHD- and relapse-free survival; GVHD, graft-versus-host disease; HLA, human leukocyte antigen; LFS, leukemia-free survival; MAC, myeloablative conditioning; MMUD, mismatched unrelated donor; MRD, matched related donor; MSD, matched sibling donor; MUD, matched unrelated donor; NA, not applicable; OS, overall survival; PBSC, peripheral blood stem cell; PBSCT, PBSC transplantation; r-ATG, rabbit ATG; RIC, reduced intensity conditioning; TBI, total body irradiation.

*Admiraal*, et al. discovered that excessive exposure to active ATG post-transplant significantly decreases the rate of successful immune reconstitution (21). In subsequent study, they revealed that for every 10% increase in the post-transplant AUC of active ATG-T, the likelihood of successful CD4+ T cell immune reconstitution decreased by 26%. Lower post-transplant active ATG exposure (< 16 AU × day/mL) and successful CD4+ immune reconstitution were both associated with improved event-free survival (72). Additionally, they found that pre-transplant active ATG exposure ≥ 40 AU × day/mL significantly reduced the incidence of grade II-IV aGVHD, cGVHD, and graft failure (21). Similarly, *Jamani*, et al. (73) from Canada discovered that the lowest quintile of pre-transplant AUC and post-transplant AUC of active ATG were associated with higher aGVHD and worse cGVHD- and relapse-free survival (cGRFS) in myeloablative allo-HSCT. A multinational prospective study by *Oostenbrink*, et al. reported that prolonged ATLG exposure (active ATLG ≥ 1 AU/mL on day +16) significantly reduced the incidence of grade II-IV aGVHD (from 50% to 8.2%) (74). A study by *Dabas*, et al. from Canada showed that high pre-transplant active ATG exposure of MNC-binding (> 282.36 UE*hr/L) and CD33+ cells- binding (> 60.53 UE*hr/L) were associated with a lower risk of relapse and better RFS. Whereas higher post-transplant exposure of lymphocyte-binding (> 1022.42 UE*hr/L) was associated with higher risk of relapse and lower RFS (75).

These studies highlight the importance of maintaining pre- or post-transplant ATG exposure within an optimal range, as both excessive and insufficient exposure compromise transplant outcomes. A Dutch retrospective analysis identified an optimal post-transplant active ATG exposure of 60–95 AU/mL/day. Sub-optimal exposure (< 60 AU/mL/day) increased grade III-IV aGVHD and NRM, while over-optimal exposure (> 95 AU/mL/day) increased relapse-related mortality (RRM). Only patients within the optimal range achieved the best 5-year event-free survival (EFS) and OS (31). A single-center prospective study from China found an optimal total active ATG exposure of 100 to 148.5 UE/mL/day in haplo-HSCT following Beijing Protocol. Interestingly, the optimal AUC group showed a significantly lower incidence of cytomegalovirus (CMV) reactivation and persistent CMV viremia compared to the non-optimal AUC group (total AUC < 100 or >148.5 UE/mL/day). While no significant difference in NRM and recurrence were observed between two groups, optimal AUC group showed a trend toward better 2-year OS (75.7% vs. 57.8%, *P* = 0.061) (19). A recent phase IV trial established an optimal post-transplant ATG exposure (55–75 AU/mL/day) for acute leukemia patients undergoing myeloablative haplo-cord HSCT. Compared to non-optimal range, patients within optimal range have lower 2-year relapse (38.9% vs. 15.2%), higher leukemia-free survival (LFS) (49.5% vs. 84.8%), superior OS (50.8% vs. 89.5%) and GRFS (39.1% vs. 71.3%), and reduced grade II-IV aGVHD (37.8% vs. 20.5%) (76).

## 7 Individualized ATG dosing strategies in allo-HSCT

### 7.1 Individualized ATG dosing guided by absolute lymphocyte count

A retrospective pharmacokinetic-pharmacodynamic study demonstrated that patients who had optimal post-transplant active ATG exposure (60–95 AU/mL/day) achieved the best 5-year OS. Further analysis of the pharmacokinetic model identified recipient’s body weight (< 50 kg) and ALC as significant covariates influencing ATG clearance. In adult allo-HSCT, conventional weight-based ATG dosing regimen achieved optimal exposure only in 30%-53% of patients (when body weight > 50 kg), whereas ALC-based dosing regimen achieved optimal exposure in 95%, thereby enhancing survival outcomes (31). Subsequently, the same team conducted a prospective single-arm Phase II study to explore the efficacy and safety of individualized ATG dosing based on ALC (36). The study identified three key parameters (recipient’s body weight, ALC before the first dose of ATG, and source of graft) to guide individualized ATG dosing (ranging from 2 to 10mg/kg). Of the 51 evaluable patients, 41 (80%) met CD4+ immune reconstitution criteria, defined as two consecutive CD4+ T cell counts > 0.05 × 10^9^/L within 100 days post-transplantation. Their previous studies have shown that patients who achieved CD4+ immune reconstitution early after transplantation had better OS, lower NRM, and fewer virus reactivations. These findings indicate that individualized ATG dosing may improve transplant outcomes by increasing the proportion of patients attaining optimal AUC (21, 72). *Seo*, et al. found that the weight-based dosing regimen in unrelated donor transplantation with reduced-intensity conditioning could cause overexposure to ATG-T in adult recipient with an ALC < 500/μl at day -7. This overexposure resulted in severe T-cell depletion, increasing the risk of life-threatening infections, and impairing OS (77). Similarly, *Woo*, et al. demonstrated in a study of adult matched sibling donor transplantation that those with an ALC < 500/μl at day -7 had a higher mortality, primarily due to infection-related complications (78). These results support adjusting the ATG dose based on ALC to avoid overexposure to ATG in patients with low ALC.

However, ALC-based individualized ATG dosing regimen may not be universally applicable. A French study enrolled 116 adult patients undergoing matched sibling or unrelated donor transplantation investigated the association between ALC before ATG administration and transplant outcomes. The study revealed that whether the ALC was higher than the median value did not affect survival (79). In a retrospective study of adult unrelated donor transplantation, *Heelan*, et al. compared weight-based dosing strategy versus ALC-guided individualized dosing strategy. The study revealed substantial dose variation between the two regimens: conventional weight-based ATG dosing yielded a median total dose of 201 mg, whereas ALC (day -2) - guided individualized dosing required a significantly higher dose with a mean of 1205 mg, representing a 5-fold increase over conventional weight-based dosing strategy. They assumed that when the administration of ATG is close to graft infusion, the lymphocytes are depleted by myeloablative conditioning, resulting in an overestimation of the ATG dose when calculated based on ALC (80).

### 7.2 Individualized ATG dosing guided by therapeutic drug monitoring

Therapeutic drug monitoring (TDM) of calcineurin inhibitors (CNIs) has been used in allo-HSCT for many years, which correlated with improved transplant outcomes (81, 82). However, current evidence regarding TDM-guided individualized ATG dosing in allo-HSCT remains limited. In a Phase II study, *Wang*, et al. developed a machine learning-based, TDM-guided individualized ATG dosing model for haplo-PBSCT. ATG was administered for 4 days (days -5 to -2) during conditioning. Active ATG concentration was detected on day -5 and -4 via flow cytometry, and the adjusted ATG doses on day -3 and -2 were calculated according to the individualized dosing model. This adjustment aimed to maintain total active ATG exposure within the optimal range of 100-148.5 UE/mL/day, a range previously identified by the same group to effectively reduce CMV/EBV reactivation in haplo-PBSCT without increasing GVHD or relapse (19, 37, 83). Additionally, researchers from the Netherlands and the United States have theoretically verified the feasibility of TDM-guided ATG dosing strategy using population pharmacokinetic model. Their TDM-guided ATG dosing framework is as follows: the total dose of ATG is administered over 4 days, and on the third day after ATG administration, the peak and trough concentrations of active ATG are measured. ATG dose on the fourth day is then adjusted according to the model-predicted AUC. If the adjustment exceeds 25% of the total ATG dose, the administration of ATG needs to be extended to the fifth day. The investigators assumed that TDM-guided ATG dosing was more accurate than ALC-guided dosing for patients presenting with immune deficiencies and/or hyperinflammation (84). A randomized phase III multicenter trial evaluated targeted ATG dosing strategy (Total ATG dose calculated based on pharmacokinetic parameters, range: 6–13 mg/kg) against a fixed dose of 10 mg/kg in adults haplo-PBSCT. Compared to fixed dosing, targeted dosing reduced CMV reactivation (54.9% vs. 31.0%), improved GRFS (48.0% vs. 63.4%), and enhanced CD4+ T-cell reconstitution (72.7% vs. 91.0%) (85).

### 7.3 Challenges in individualized ATG dosing in allo-HSCT

**Figure 3:**
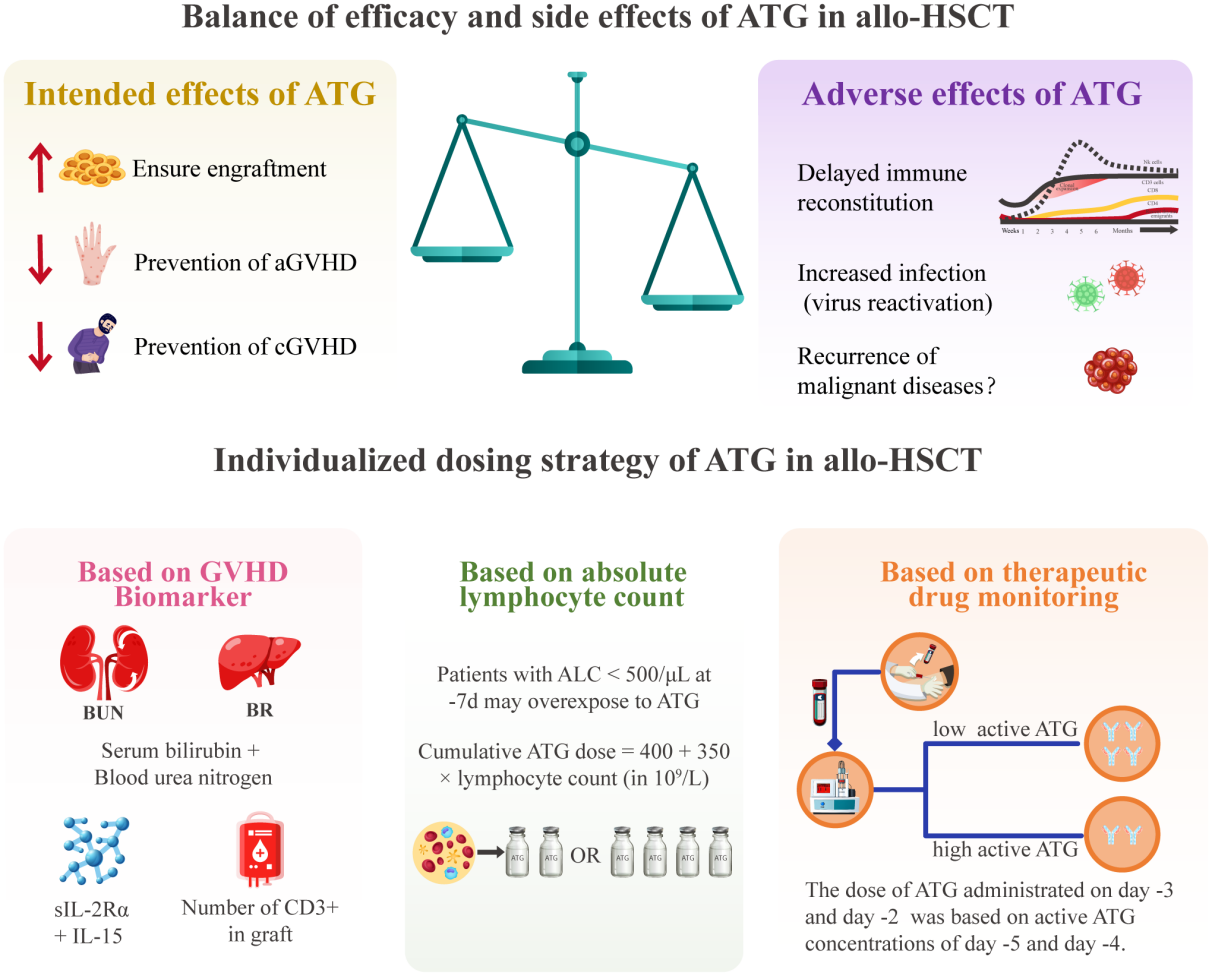
The balance of efficacy and toxicity of ATG and individualized dosing strategies in allo-HSCT. aGVHD, acute graft-versus-host disease; allo-HSCT, allogeneic hematopoietic stem cell transplantation; ALC, absolute lymphocyte count; ATG, anti-thymocyte globulin; BUN, blood urea nitrogen; CD, cluster of differentiation; cGVHD, chronic graft-versus-host disease; GVHD, graft-versus-host disease; IL, interleukin; slL-2Ra, soluble interleukin-2 receptor alpha.

It should be noted that individualized ATG dosing in allo-HSCT faces significant challenges. First, detecting active ATG is complex and difficult to standardize. Flow cytometry, the predominant detection method for active ATG, demonstrates an inter-laboratory variability due to heterogeneity in flow cytometer models and biological materials (e.g., cells and antibody clones). Second, the clinical assessment of optimal ATG exposure lacks consensus criteria. Different optimal ATG exposure ranges were reported across centers due to inconsistent optimal exposure definitions [(e.g., successful CD4+ T cell reconstitution (31, 86), or reduction of virus reactivation (83)]. Heterogeneity in the timing and dosing of ATG administration further complicates this issue, and collaborative efforts are needed to establish a consensus-defined optimal active ATG exposure in allo-HSCT. Third, current personalized dosing strategies including ALC-guided and TDM-guided approaches, have population-specific limitations (14, 84, 87, 88) (**Figure 3**). It is necessary to conduct further research to establish a universally applicable individualized dosing regimen using population pharmacokinetic modeling.

## 8 Discussion

This review discussed the challenges of optimizing ATG dosing in allo-HSCT to balance GVHD prophylaxis with immune reconstitution, while minimizing malignant disease recurrence and life-threatening infections. Extensive research has focused on weight-based ATG dosing regimens in allo-HSCT, yet this approach remains suboptimal in addressing the pharmacokinetic variability mediated by multiple parameters, including genetic polymorphisms (e.g., HLA compatibility and Fcγ receptor genotypes), timing of ATG, anti-ATG antibody development, and comorbidities. The weight-based ATG dosing approach failed to address the substantial pharmacokinetic variability among patients, thus attempting to establish body weight based optimal ATG dosing regimen will continue to prove futile. Pharmacodynamic studies demonstrated that lower ATG concentration was associated with increased risks of aGVHD and cGVHD, although its impact on TRM and relapse remains unclear (47, 62, 63). Notably, emerging evidence highlights the association between ATG exposure and transplant outcomes such as GVHD incidence, immune reconstitution, relapse, and OS (21, 31, 72, 75). Importantly, an optimal ATG exposure range has been identified, associated with reduced viral reactivation, accelerated immune reconstitution, and improved OS (19, 31).

TDM and pharmacogenomics (PGx) are fundamental approaches for achieving personalized dosing in clinical practice. Advances in understanding ATG-PGx, including drug-metabolizing enzymes, therapeutic targets, and drug transporters, will enable optimized balancing of ATG’s efficacy against treatment-related toxicity. Integrating TDM with PGx in ATG personalized dosing represents a promising strategy to improve outcomes of allo-HSCT. Recent studies have shown promising outcomes using individualized ATG dosing strategies based on ALC or TDM (36, 37). However, current individualized ATG dosing protocols are often derived from physiologically based pharmacokinetic (PBPK) and population pharmacokinetic (popPK) models (21, 56). These protocols exhibit inherent static limitations of failing to integrate real-time patient data and dynamic health trends. Model-informed precision dosing (MIPD) provides a potential solution for optimizing ATG dosing via mathematical modeling that integrates multidimensional data, including patient characteristics, drug properties, and disease status. Collaboration across clinicians, informaticians, clinical pharmacologists, and TDM specialists will establish ethical framework for data sharing, technology accessibility, and patient privacy, thereby facilitating clinical implementation of MIPD. Artificial intelligence (AI) and machine learning (ML) represent emerging tools for advancing MIPD in personalized medicine, but their clinical application remains experimental with unproven benefits for patient care (89–91). Robust clinical validation and technological innovation are essential to overcome inherent challenges, including data privacy and algorithmic bias, thereby enabling tangible patient benefits and facilitating clinical implementation (92). In conclusion, the ongoing development and optimization of individualized ATG dosing strategies are critical for enhancing the safety and efficacy of allo-HSCT, ultimately improving transplant outcomes.
